# Editorial: SOTRAP – A Journal on the Rise

**DOI:** 10.5964/sotrap.18985

**Published:** 2025-07-28

**Authors:** Daniel Turner, Ross Bartels, Sarah Beggs Christofferson, Sonja Etzler, L. Maaike Helmus, Julia Sauter, Martin Rettenberger

**Affiliations:** 1 University Medical Center Mainz, Mainz, Germany; 2 University of Lincoln, Lincoln, United Kingdom; 3 University of Canterbury, Christchurch, New Zealand; 4 Medical Center – University of Freiburg, Freiburg, Germany; 5 Simon Fraser University, Burnaby, Canada; 6 University of Kassel, Kassel, Germany; 7 Centre for Criminology (Kriminologische Zentralstelle – KrimZ), Wiesbaden, Germany

Sexual Offending: Theory, Research, and Prevention (SOTRAP) is the official publication of the International Association for the Treatment of Sexual Offenders (IATSO). The journal primarily publishes works that enhance or illuminate relevant clinical practice, science, and policy regarding the etiology, prevention, (risk) assessment, treatment, and management of individuals who have committed sexual offenses or are at risk of doing so. Additionally, SOTRAP welcomes contributions addressing topics in sexual science that are not directly related to individuals who have committed sexual offenses.

SOTRAP emerged in 2020 as the successor to the journal „Sexual Offender Treatment“ (SOT). The founding members of SOTRAP included Martin Rettenberger, L. Maaike Helmus, Mark E. Olver, and Reinhard Eher. While Martin and Maaike continue to serve as editors of SOTRAP, Mark and Reinhard have since stepped down from their positions. Nevertheless, we would like to take this opportunity to thank Mark and Reinhard for their pioneering work with the journal, as SOTRAP would certainly not be where it is today without their considerable efforts.

With the change in name, a transition to a new publication platform also took place, and since 2020, SOTRAP has been part of the PsychOpen GOLD family under the Leibniz Institute for Psychology (ZPID) which is located in Germany. PsychOpen GOLD journals enable authors to publish open access at no cost, representing a unique and innovative model in the scientific community. To remain part of PsychOpen GOLD, the quality and reach of SOTRAP are regularly re-evaluated. We would therefore like to thank all authors who have published their outstanding work in SOTRAP over the past five years, and simultaneously encourage authors to consider submitting their work to SOTRAP in the future. To date, all evaluations have been overwhelmingly positive, and SOTRAP has undergone a highly favorable development in recent years. In the following, we wish to inform you about the most important developments and innovations.

## Steadily Increasing Submissions and Reach

While a total of 13 articles were submitted in SOTRAP’s inaugural year, the number of submissions reached a new high of 36 articles last year. This positive trend has continued in the current year. At the same time, our articles are increasingly being recognized by a broad scientific community, with the articles published so far this year receiving over 2,000 views within just a few days. This demonstrates that SOTRAP’s readership extends far beyond IATSO members and that the journal has now established itself as an internationally recognized publication. Consequently, the number of citations per article has also been steadily increasing.

Since 2023, SOTRAP has been indexed in Scopus. We began in 2023 with a CiteScore of 0.5 (number of citations over the past four years divided by the number of documents published in the same period). By 2024, the CiteScore had already risen to 1.7. Furthermore, after a multi-month application process, we achieved inclusion in PubMed Central (PMC), which has further significantly increased SOTRAP’s reach. We plan to apply for inclusion in Web of Science in 2026, which will allow SOTRAP to receive an Impact Factor in the future.

## Expansion of the Editorial Team

Due to the increasing number of publications, the original editorial team was no longer able to manage the resulting workload alone. We are therefore grateful that, in recent years, we have succeeded in gradually expanding the editorial team and competently compensating for the departures of Mark and Reinhard. Our current editorial team now comprises seven members. In addition to the two Editors-in-Chief, Martin Rettenberger (Kriminologische Zentralstelle, Wiesbaden, Germany) and Daniel Turner (University Medical Center Mainz, Germany), the team includes Ross Bartels (University of Lincoln, UK), Sarah Beggs Christofferson (University of Canterbury, Christchurch, New Zealand), Sonja Etzler (University Medical Center Freiburg, Germany), L. Maaike Helmus (Simon Fraser University, Burnaby, Canada), and Julia Sauter (University of Kassel, Germany). This constitutes an international, young, and dynamic team, and our collaboration is a source of great satisfaction for all of us.

SOTRAP is now characterized by a wealth of outstanding publications. To thematically cluster submitted works, the editors have decided to introduce so-called Thematic Sections, which rotate every two years. A Thematic Section represents a collection of articles on a specific overarching topic of interest. In 2024/2025, our Thematic Section was titled “Paraphilias and Sexual Offending” and currently includes three articles, with three more already accepted for publication. The current Thematic Section encompasses a diverse range of articles, such as a contribution by Brooke Davis et al. on Religion, Sadism, and Masochism, or a highly interesting theoretical article by Hannah Stewart and Mary Ann Campbell discussing a biopsychosocial model for the etiology of sexual interest in children. We would particularly like to highlight that the organization of Thematic Sections is generally entrusted to an external Guest Editor. We therefore extend our sincere thanks to Skye Stephens, who, together with Maaike, was responsible for the Thematic Section “Paraphilias and Sexual Offending” and did an outstanding job.

Furthermore, it is important to emphasize that SOTRAP is an international journal, and we are committed to representing research from a wide variety of countries. To better achieve this goal, we have created a new article category: the so-called Country Reports. In addition to works on innovative national approaches to the assessment, management, and treatment of individuals who have committed sexually motivated violence or are at risk of doing so, this category also welcomes submissions on political, juridical, and public policy-related issues. We explicitly encourage authors from countries that otherwise receive less attention in the scientific community to submit articles for this category. For example, we have already published two articles by Jacob Mensah Agboli on the management of sexual offenses in Ghana.

## Future Developments and Opportunities for Your Involvement

For 2026, we aim to make our journal even more diverse and inclusive, thereby better representing the entire membership of IATSO. As a first step, we intend to expand our Editorial Board by recruiting new members. Membership on the Editorial Board will provide the opportunity to help shape SOTRAP’s future and influence its further development. Conversely, we would appreciate it if all Editorial Board members could occasionally review submitted manuscripts. Should you be interested in joining the Editorial Board, please submit an application including a brief curriculum vitae and your most important publications to editors@sotrap.psychopen.eu. We particularly encourage people from South America, Africa, and Asia, as well as those at the early stages of their academic careers, to apply for membership on the Editorial Board.

Additionally, there is currently no Thematic Section planned for 2026/2027. Therefore, we invite you to submit proposals for future Thematic Sections and, in doing so, to serve as Guest Editor for the section. If you are interested in actively shaping SOTRAP in this capacity, please contact us at editors@sotrap.psychopen.eu.

In conclusion, we would like to thank all those who have supported and shaped SOTRAP and IATSO through their active participation in recent years. We are convinced that SOTRAP and IATSO offer a unique opportunity for fruitful and stimulating exchange.



**Daniel Turner, Ross Bartels, Sarah Beggs Christofferson, Sonja Etzler, L. Maaike Helmus, Julia Sauter, & Martin Rettenberger**
*(Editors-in-Chief)*
 
 



